# An *ex vivo* cornea infection model

**DOI:** 10.1016/j.mex.2020.100876

**Published:** 2020-04-07

**Authors:** Uloma Ubani-Ukoma, Anuj Chauhan, Gregory Schultz, Daniel J. Gibson

**Affiliations:** a Department of Pharmaceutics & Pharmaceutical Technology, Faculty of Pharmacy, University of Lagos, Nigeria; b Department of Chemical and Biological Engineering, Colorado School of Mines, United States; c Institute for Wound Research, Department of Obstetrics and Gynecology, University of Florida, Gainesville, United States

**Keywords:** Bacterial keratitis, Native matrix models, Ocular drug delivery, *Ex vivo* tissue models

## Abstract

In vitro screening and testing of drugs and devices is necessary, but in vitro conditions differ greatly from those found in vivo. These differences can lead to false promises of efficacy, or can hide problems of tissue compatibility. Models with *ex vivo* tissues can be highly valuable bridges which provide relevant matrices for testing [1], [2], [3], [4], [5], [6], [7], [8], [9]. *Ex vivo* tissue models which are closer both biochemically and biophysically can provide useful feedback in a more time- and cost-efficient manner. Herein we describe an *ex vivo* corneal model for use in drug delivery testing and corneal infection modeling [10]. The protocol covers the tissue harvesting, sterilization, inoculation, and bacterial load quantification. We envision that the model can be used to study bacterial physiology on metabolizable matrices and to study the direct effects of microbial colonization on the cornea's integrity and clarity.

• Devitalized cornea.
• Non-submersed conditions.
• Contact lens compatible.

## Graphical abstract

**Figure:**
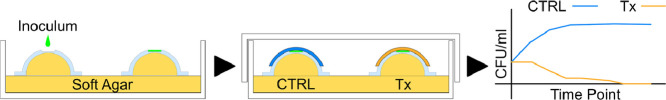

**Table:** Specifications Table

Subject Area	Immunology and Microbiology
More specific subject area	Ocular Drug Delivery
Method name	*Ex vivo* corneal infection model
Name and reference of original method	Berkowski, W. M. et al. Assessment of Topical Therapies for Improving the Optical Clarity Following Stromal Wounding in a Novel *Ex Vivo* Canine Cornea Model. Invest Ophthalmol Vis Sci 59, 5509-5521, doi:10.1167/iovs.17-23,085 (2018).
Resource availability	If applicable, include links to resources necessary to reproduce the method (e.g., data, software, hardware, reagent) 1 cm x 1 cm cuvette spectrophotometer set to 640 nm. 1 cm x 1 cm cuvette. 10% povidone iodine (PI). 15 ml tubes containing 7 ml PBS-T. 18.2 MΩ deionized water or distilled deionized water. 2 pairs of forceps. A white light transilluminator or a digital automated colony counter. Air tight container; 15- or 50-ml conical tube will suffice. Autoclave. Camera to document corneal integrity. Centrifuge. Clean 1200 ml glass flask. Fluconazole. Frozen aliquot of pathogen of interest. gDNA 16S rRNA sequencing service Granulated agar. Hot plate with magnetic stirrer. Humidified incubator set to 37 °C. Magnetic stir bar. One sterile 15 ml conical tube for each cornea. 5 ml of TSB for each tube. The tube should have visible volume gradations. Orbital shaker set to 37 °C at 150 rpm. Phosphate buffered saline at pH 7.4 (PBS, w/o Mg or Ca) Phosphate buffered saline at pH 7.4 with 0.001% v/v Tween 80 (PBS-T). Sonication bath. Sterile 12 mm punch biopsies. Sterile 15 ml conical tubes. Sterile 250 ml glass flask with 20–30 ml of TSB. Sterile 50 ml tubes. Sterile 8 mm punch biopsies, 8 mm trephine, or 8 mm vacuum trephine. Sterile 90 mm Petri dishes. Sterile 90 mm soft agar plates with 21.25 µg/ml fluconazole. Sterile 90 mm tryptic soy agar plates. Sterile field in a biosafe laminar flow hood. Sterile mold, we used a porcelain one. Sterile scalpel. Sterile soft agar Sterile swabs, rods, beads, or sticks for streaking plates. Sterile tubes for 10-fold serial dilution series. Tenotomy scissors. Timer. Tryptic soy broth (TSB). Tube vortexer. Tubes for serial dilution prior to plating for bacterial counts. White light transilluminator for colony counting. Whole frozen rabbit eyes. Optional: Automated digital colony counter. Optional: Cotton gauze. Optional: Crescent knife or blunted 18–23 G needle. Optional: Dissecting *“t”*-pins. Optional: Interscience easySpiral Dilute^Ⓡ^ automatic serial diluter and plater. Optional: Polystyrene 15 ml tube tray. Optional: Series of test sterilants at test concentrations.

## Method details

IMPORTANT: All steps within this method should be carried out using aseptic technique in a biosafe hood.

### Preparation of Soft Agar, Tryptic Soy Agar (TSA) and Tryptic Soy Broth (TSB)

#### Materials

• Tryptic soy broth.
• Granulated agar (Fisher Scientific).
• 18.2 MΩ deionized water or distilled deionized water.
• Clean 1200 ml glass flask.
• Magnetic stir bar.
• Hot plate with magnetic stirrer.
• Autoclave.
• Fluconazole (Fisher Scientific, Tocris Bioscience).
• Sterile 90 mm Petri dishes.

#### Method

The soft agar used in this protocol is solely for physical support, not a growth medium; it is expected to be sufficient for any pathogen chosen. Soft agar plates were made by dissolving 18 g of TSB and 3 g of granulated agar in 600 ml 18.2 MΩ deionized water (Milli-Q water) with stirring using the magnetic stirrer. Tryptic Soy Agar (TSA) plates were made by dissolving 24 g of TSA powder in 600 ml of Milli-Q water and mixed using a magnetic stirrer plate. Tryptic Soy Broth (TSB) was prepared by dissolving 18 g of TSB powder in 600 ml of Milli-Q water. All preparations were autoclaved at liquid cycle for 30 min or 45 min at 121 °C. To prevent fungal growth during the three-day study, fluconazole 21.25 µg/ml was added to the soft agar after autoclaving before pouring into sterile 90 mm Petri dishes.

### Preparation of bacterial culture

#### Materials

• Sterile field in a biosafe laminar flow hood.
• Frozen aliquot of pathogen of interest.
• TSA plates.
• Humidified incubator set to 37 °C.
• Sterile 15 ml conical tubes.
• Tryptic soy broth (TSB).
• Orbital shaker set to 37 °C and 150 rpm.
• Sterile 250 ml glass flask with 20–30 ml of TSB.
• Sterile swabs, rods, beads, or sticks for streaking plates.
• Sterile 90 mm tryptic soy agar plates.
• 1 cm x 1 cm cuvette.
• 1 cm x 1 cm cuvette spectrophotometer set to 640 nm.

#### Method

The bacterial culture conditions described are for the two pathogens we have chosen to test, *Pseudomonas aeruginosa* (PAO1) and S*taphylococcus aureus* (SA35556); other pathogens or strains might require different protocols to obtain the desired inoculum. Each pathogen was individually streaked for isolation on TSA plates from frozen stock cultures and incubated for 16–18 h at 37 °C. A 15 ml culture tube containing 5 ml of sterile TSB was inoculated with a single colony of the pathogen, vortexed and placed in an orbital shaker at 150 rpm and 37 °C overnight. About 0.2–0.5 ml bacterial culture was taken from the overnight tube and added to a 250 ml sterile flat-bottom flask containing 20–30 ml TSB. The conical flask was placed in the shaker for 2 h or until an optical density of 0.2–0.4 corresponding to 10^8^ CFU/ml was obtained at 640 nm with a standard laboratory spectrophotometer with a 1 cm pathlength cuvette.

### Preparation of the *ex vivo* rabbit corneas

#### Materials

• Sterile field in a biosafe laminar flow hood.
• Whole frozen rabbit eyes.
• *Optional:* Polystyrene 15 ml tube tray.
• *Optional:* Dissecting *“t”*-pins.
• *Optional:* Cotton gauze.
• Sterile 8 mm punch biopsies, 8 mm trephine, or 8 mm vacuum trephine.
• 2 pairs of forceps.
• *Optional:* Crescent knife or blunted 18–23 G needle.
• Tenotomy scissors.
• Sterile scalpel.
• Sterile 90 mm petri dish.
• Air tight container; 15- or 50-ml conical tube will suffice.

#### Method

**Fig. 1:**
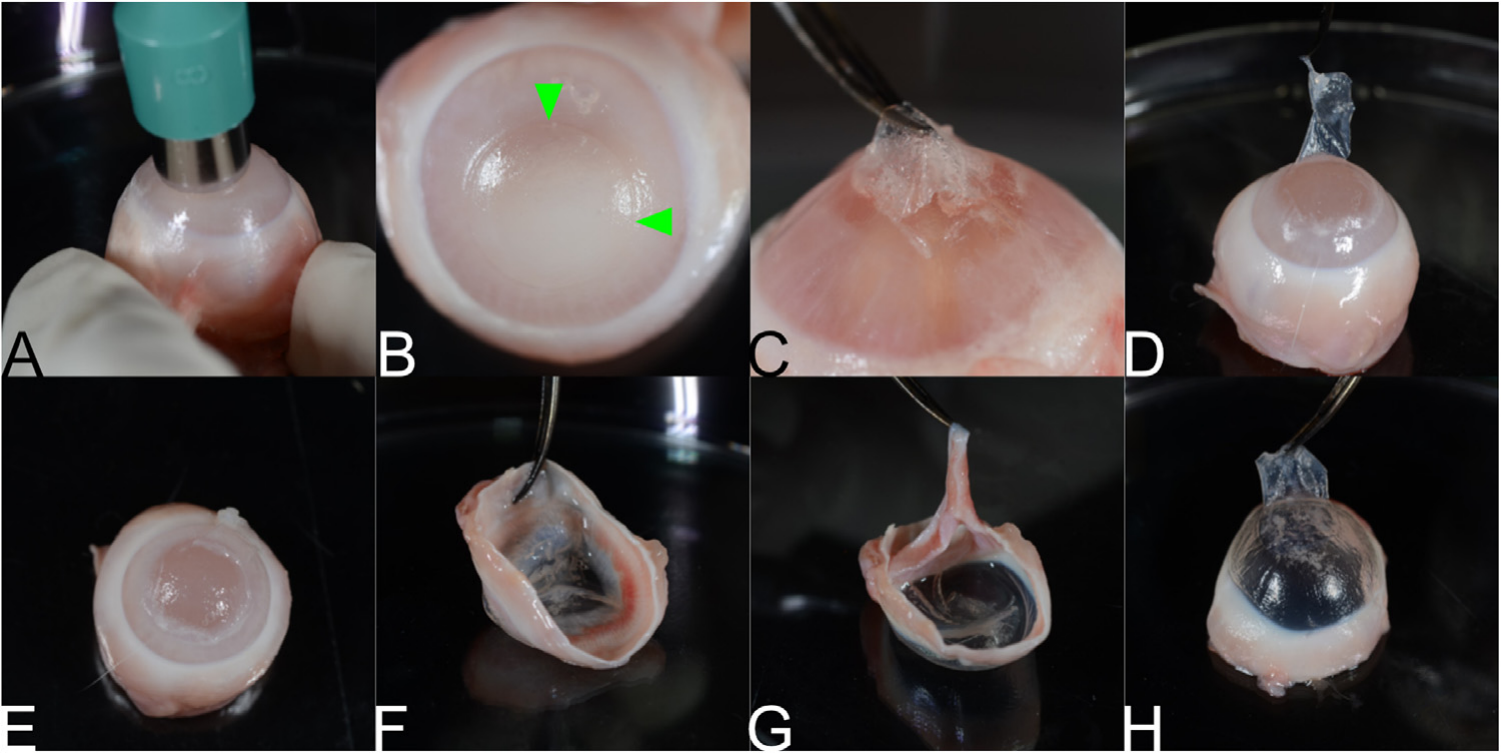
(A) A frozen rabbit eye. (B) Biopsy punch placed on the eye with slight pressure. (C) The eye showing the cut outline made by the punch. (D) Removal of epithelium and superficial stroma. (E) Exposure of stroma with partially removed epithelium. (F) Exposed stroma with flap. G) Excised cornea with iris. (H) Removal of the iris and ciliary body. (I) Corneoscleral button showing removed epithelium, superficial stroma and iris (flap was excised for all corneas used).

Whole frozen rabbit eyes (globes) can be obtained from an abattoir (Pel Freeze, Rogers, AK, USA) or from research collaborators; it is best begin with the eyes fully frozen. The frozen eye should be held firmly via either a gloved hand (Fig. 1(A)), or a polystyrene tray from 15 mm conical tubes and dissection T-pins (not shown). For the polystyrene tray-based approach, place absorbent materials such as gauze into the “wells” that hold the tubes until the eye can be placed such that ~1/2 of the globe is above the surface. If the eye still moves too much, dissecting pins can be placed through the residual conjunctiva or muscle tissue into the polystyrene tray. For this particular model, the stroma is the targeted growth media. To expose the stroma, a lamellar keratectomy (LK) is performed. The globe should still be mostly frozen at this point; its rigidity makes the process easier. A gloved thumb or finger can be used to transfer some heat to the cornea so that it is not entirely frozen. A sterile 8 mm punch biopsy, a trephine, or vacuum trephine can be used to create a partial thickness wound in the center of the cornea (Fig. 1(B)). A crescent knife, forceps, or a blunted needle can then be used to dissect the corneal button away from the globe (Fig. 1(D)). Any remaining attachments can be cut with either curved tenotomy scissors or a crescent knife (Fig. 1(E)). Then, the cornea and a scleral rim are excised with a sterile disposable scalpel (Fig. 1(F) and (G)). Finally, the iris and ciliary body are removed with forceps (Fig. 1(H)) by forcible peeling. This is a stopping point and the corneas can be stored in an airtight container at −20 °C until needed.

### The confirmed sterilization method

#### Materials

• Sterile field in a biosafe laminar flow hood.
• 10% povidone iodine (PI).
• 6 prepared corneas from “Preparation of the *ex vivo* rabbit corneas” for each experiment group or control.
• Sterile 50 ml tubes.
• Camera to document corneal integrity.
• Timer.
• Phosphate buffered saline at pH 7.4

#### Method

Groups of 6 corneas are placed in 50 ml tubes and submerged in 35 ml of 10% PI for 30 min at room temperature. The PI is decanted and replaced with 35 ml of sterile PBS at least 5 times or until all visible evidence of PI is gone. These corneas should be immediately used in the next step, no storage testing has been performed to affirm the duration of sterility.

### Infection of *ex vivo* rabbit corneas

#### Materials

• Sterile field in a biosafe laminar flow hood.
• Sterilized injured rabbit corneas; with at least 6 replicates per group per time point.
• Sterile mold, we a porcelain one here, but polytetrafluoroethylene (PTFG, Teflon™) blocks (Brightbill Corneal Cutting Block, Storz Ophthalmic Instruments), custom 3D printed acrylonitrile butadiene styrene (ABS), and dental impression materials such as polydimethylsiloxane (PDMS) have been used for this and related purposes.
• Sterile soft agar
• Sterile 90 mm soft agar plates with 21.25 µg/ml fluconazole.
• Sterile 12 mm punch biopsies.
• Phosphate buffered saline at pH 7.4 with 0.001% v/v Tween 80 (PBS-T).
• 15 ml tubes containing 7 ml PBS-T.
• Sonication bath.
• Tube vortexer.
• Sterile tubes for 10-fold serial dilution series.
• Sterile swabs, rods, beads, or sticks for streaking plates.
• Sterile 90 mm tryptic soy agar plates.
• Humidified incubator set to 37 °C.
• White light transilluminator for colony counting.
• *Optional:* Interscience easySpiral Dilute^Ⓡ^ automatic serial diluter and plater.
• *Optional:* Automated digital colony counter.

#### Method

**Fig. 2:**
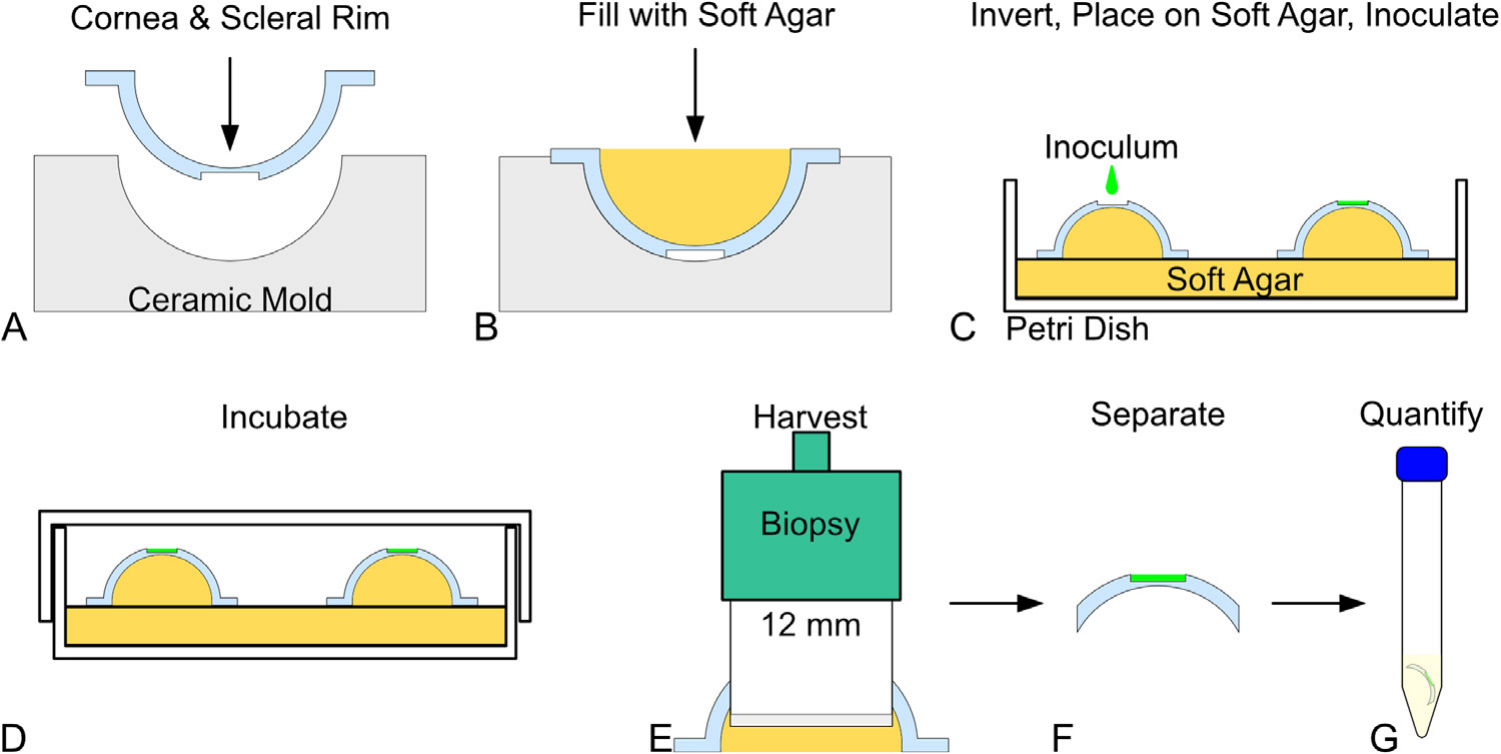
A visual outline of the model. (A) The sterilized cornea is placed into a supporting mold, and (B) is filled with soft agar. (C) The agar-filed cornea is placed on a dish with soft agar and inoculated. (D) The inoculum is allowed to establish for a duration dependent upon the experiment, and the experiment is then performed. (E) At each time point, the contact lens is removed and the cornea is collected by biopsy. (F) The agar is removed and (G) the cornea is assayed for total viable bacteria.

The corneas are placed in a sterile mold, epithelial side down (Fig. 2(A)), and filled with sterile soft agar (Fig. 2(B)), and left to stand until the agar solidified. This is typically about 10 min, but can be affirmed by gently touching the soft agar surface with a sterile pipette tip or swab. The corneas with solidified agar were then placed epithelial side up on sterile soft agar plates with up to 3 corneas per plate (Fig. 2(C)). The site of injury is inoculated with 1 µl of pathogen (10^8^ CFU/ml) or 1 µl of PBS (Fig. 2(C)). The lid is placed back on the dish and all dishes are incubated at 37 °C (Fig. 2(D)) for at least 2 h for an acute infection model (as described herein). The duration used depends upon the end-user's experimental goal, and longer incubations can be used for more established colonization as we have done in porcine skin wound models [3], [4], [5]. The level of inoculation can also be varied depending on the end-user's needs.

The experimental contact lenses or other topical treatments are applied per the experimental design within a biosafe hood. The lid should only be removed within a sterile biosafe hood, or else contamination is certain. At each time point, the contact lenses are removed and the injured sites and surrounding cornea are collected with sterile 12 mm punch biopsies (Fig. 2(E)). The agar is peeled away from each corneal button (Fig. 2(F)), and the buttons are individually transferred into separate sterile 15 ml tubes containing 7 ml PBS-T (Fig. 2(G)). Each tube is vortexed multiple times and the resulting suspension is serially diluted at least 4 × 10-fold steps and plated on TSA plates using either common streaking techniques or a spiral plater. TSA plates were incubated for 16–18 h at 37 °C and colonies are counted to determine the viable bacterial cell count in CFU/ml. The time line of CFU/ml for each group is the basis for experimental comparison [10]. Additional analysis can be performed to inform the nature or limit of effects seen. Images of the corneas and surrounding area are a relatively simple method to monitor the spread of bacteria. Standard hematoxylin & eosin stained sections can enable the monitoring of depth effect.

### Sterilization screening for alternative disinfectants

If the sterilization method above is incompatible with an experiment, the following screening procedure can be used to screen other agents, doses, and conditions.

#### Materials

• Sterile field in a biosafe laminar flow hood.
• 10% povidone iodine (PI).
• *Optional:* Series of test sterilants at test concentrations.
• 5 prepared and sterilized corneas for each group.
• Sterile 50 ml tubes.
• Camera to document corneal integrity.
• Timer.
• Phosphate buffered saline at pH 7.4 (PBS)
• PBS with 0.001% v/v Tween 80 (PBS-T)
• One sterile 15 ml conical tube for each cornea. 5 ml of TSB for each tube. The tube should have visible volume gradations.
• Orbital shaker set to 37 °C at 150 rpm.
• 1 cm x 1 cm cuvette.
• 1 cm x 1 cm cuvette spectrophotometer set to 640 nm.
• Centrifuge.
• gDNA 16S rRNA sequencing service
• Sterile 90 mm tryptic soy agar plates.
• Tubes for serial dilution prior to plating for bacterial counts.
• Sterile swabs, rods, beads, or sticks for streaking plates. Alternatively, use a spiral plater.
• A white light transilluminator or a digital automated colony counter.

#### Method

**Fig. 3:**
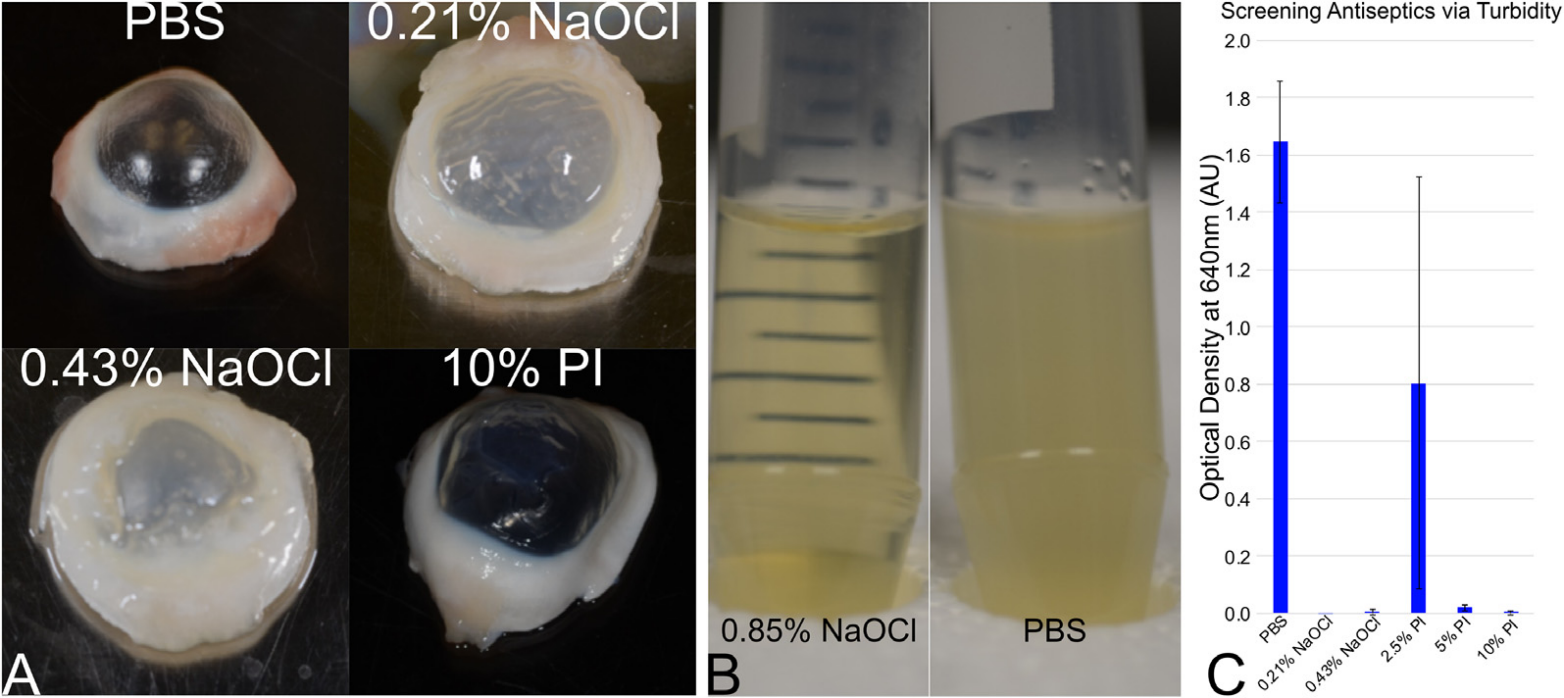
Screening sterilization techniques. A large number of potential sterilants can be assessed rapidly via (A) a gross assessment of tissue toleration. (B) The efficacy can be grossly assessed by observable turbidity after incubating treated corneas in growth medium. Solutions appearing to be grossly effective can be further assessed quantitatively by measuring (C) turbidity via spectrophotometry.

The excised corneas are subjected to different chemical antiseptics to determine the appropriate sterilization technique. A rapid screening approach is first used to screen potential candidates and concentrations. First, groups of 5 corneas were placed in 50 ml tubes and submerged in 35 ml of antiseptic for 30 min at room temperature. A negative control group is placed in 35 ml of PBS for 30 min and a positive control group is placed in 35 ml of 0.85% NaOCl for 30 min, both at room temperature. The antiseptic is decanted and replaced with 35 ml of sterile PBS and the tube is then vortexed to rinse the cornea. Repeat the rinsing and vortexing with fresh PBS at least 5 times or until all visible evidence of the antiseptic solution was gone. Next, the rinsed corneas are then individually imaged to determine tissue integrity (Fig. 3(A)); agents with extensive damage can be removed from further screening. For the purpose of demonstration, the NaOCl samples are further studied herein. The corneas are then individually placed into a 15 ml tube containing 5 ml of TSB, and the tubes are placed in a shaker overnight at 37 °C at 150 rpm. The next day the turbidity was checked via a gross method to determine if the tube's lines could be perceived through the broth (Fig. 3(B), positive vs. negative control). The broth's turbidity was then quantified via spectrometry (Fig. 3(C)). While some of the agents had a detrimental impact on the cornea (Fig. 3(A)), others have found that it can be a combination of agent and duration of exposure [4]. Further refinement and adjustment can be pursued if a desired agent does not pass the first screening.

For solutions which do not grossly affect the cornea's integrity, and effectively sterilized the corneas, a final test is performed to ensure that bacteria can colonize and grow on the tissue. The corneas are placed face down in a sterile mold and filled with agar prior to being inoculated (Fig. 2). The inoculum volume, CFU/ml, and incubation conditions might vary for each pathogen, but we started with between 1 and 5 µl of pathogen at 10^8^ CFU/ml, and incubated for 16 h at 37 °C. After incubation, the infected injury and peripheral cornea are collected by a 12 mm punch biopsy, and the corneal buttons are separated from the agar and individually submerged in 7 ml PBS-T. The samples are vortexed 5 times and the tubes placed in a sonication bath for 90 s intermittently, at 60 s intervals, for 5 cycles. The resultant suspension is centrifuged using Eppendorf Centrifuge 5424 (Hamburg, Germany) and the gDNA of the sediment is collected and submitted for bacterial identification by 16S rRNA sequencing to ensure that the identity of bacteria matched that of the inoculum [11]. An example of results from a set of tested conditions is presented in Table 1.

**Table 1:** Result of 16S rRNA genetic sequencing of infected corneas.

Decontamination technique	Level of identification%	Contaminating organism
10% Povidone Iodine	99	None
5% Povidone Iodine	90	None
10% Povidone Iodine and soaking in TSB	83	*Pseudomonas* spp, uncultured bacterium clone, *Xanthomonas* spp, *Cyanobacterium* spp, *Calothrix* spp, etc

**Fig. 4:**
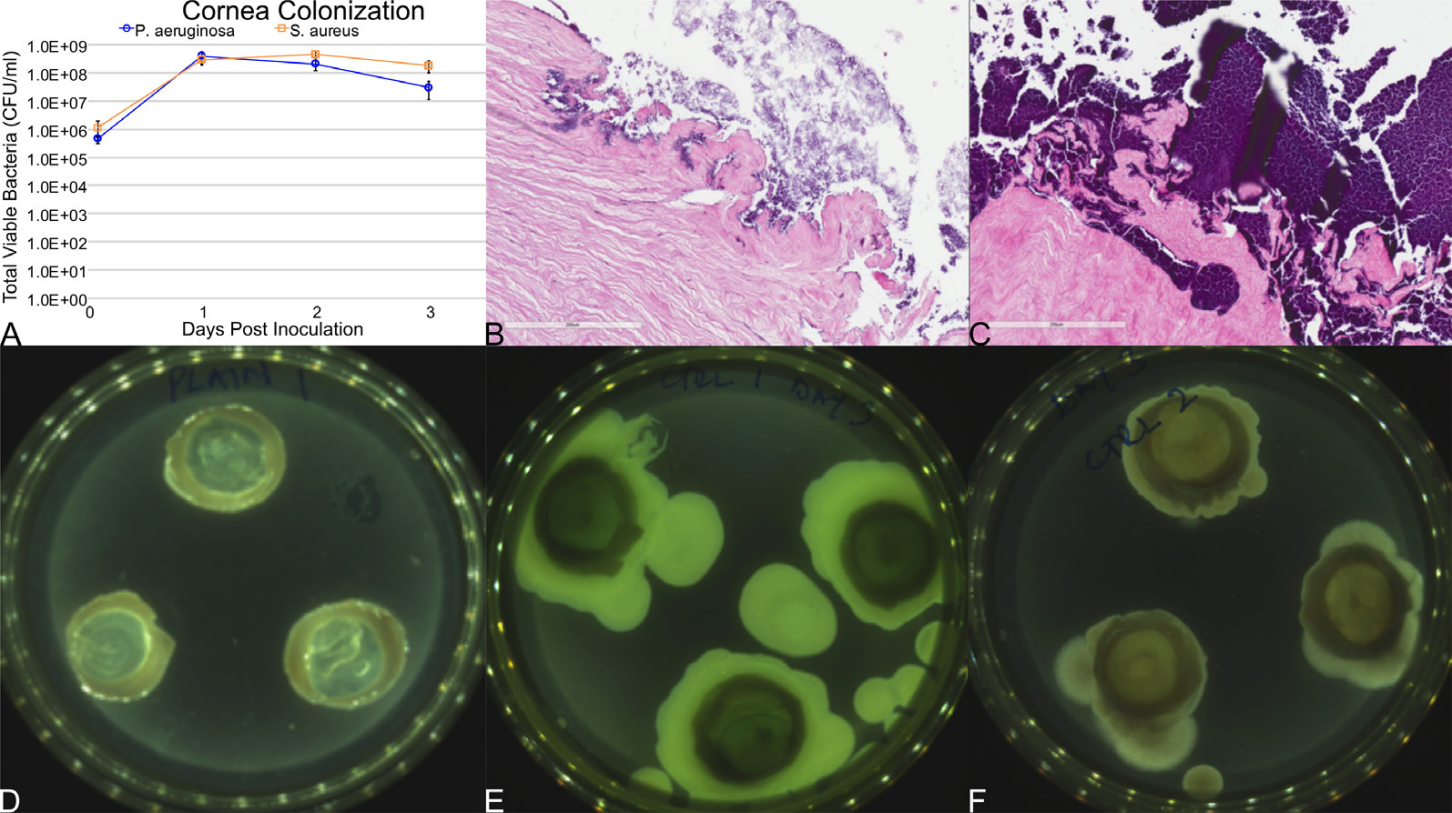
Example of successful colonization. (A) Viable CFU/ml for both *P. aeruginosa* (PAO1) and *S. aureus* (SA35556). H&E can be used to visualize (B) *P. aeruginosa* (PAO1) and (C) *S. aureus* (SA35556). (D) An image of a clean cornea vs. one infected with (E) *P. aeruginosa* (PAO1) or F) *S. aureus* (SA35556).

An example of the growth of *P. aeruginosa* (PAO1) and *S. aureus* (SA35556) is shown (Fig. 4). While the growth in the central 12 mm of the cornea might plateau numerically (Fig. 4(A)), there might be changes in the penetration (Fig. 4(B) and (C)), or the lateral spread of the bacteria over the surface (Fig. 4(D) and (F)). The inoculum which provides the desired bacterial load and penetration depth should be used in the experimental use of the model.
